# miR-205 promotes proliferation and invasion of laryngeal squamous cell carcinoma by suppressing CDK2AP1 expression

**DOI:** 10.1186/s40659-015-0052-5

**Published:** 2015-10-29

**Authors:** Gang Zhong, Xingao Xiong

**Affiliations:** Department of Hematology, Wuhan Union Hospital of Tongji Medical College, Huazhong University of Science and Technology, Wuhan , China; Department of Otolaryngology, Wuhan Union Hospital of Tongji Medical College, Huazhong University of Science and Technology, 1277 Jiefang Avenue, Wuhan, Hubei 430022 China

**Keywords:** miR-205, Laryngeal squamous cell carcinoma (LSCC), CDK2AP1, *c-Myc*, *CyclinD1*

## Abstract

**Background:**

The aberrant expression of microRNAs (miRNAs) has been found in various types of cancer. miR-205 was reported to be upregulated in laryngeal squamous cell carcinoma (LSCC) tissues, however, the mechanisms by which miR-205 functions as a regulator of LSCC are largely unknown.

**Results:**

In this study, Real-time qPCR and Western blot assay showed that expression of miR-205 was upregulated and expression of cyclin-dependent kinase 2-associated protein 1 (CDK2AP1) was downregulated in LSCC tissues. The expression levels of miR-205 were negatively related to those of CDK2AP1 in LSCC tissues and cell lines. Moreover, we found that miR-205 was the upstream regulator of CDK2AP1 and could suppress the CDK2AP1 expression in LSCC cells. 3-(4,5-dimethylthiazal-2-yl)-2,5-diphenyl-tetrazolium bromide assays and transwell invasion assay were performed to test the proliferation and invasion of LSCC cells. Gelatin zymography was used to detect the activity of MMP2 and MMP9. CDK2AP1, c-Myc and CyclinD1 expression in cells was assessed with Western blotting. We found that miR-205 was the upstream regulator of CDK2AP1 and could suppress the expression of CDK2AP1 in LSCC cells. In addition, miR-205 significantly induced cell proliferation and invasion by suppressing CDK2AP1 expression. Consistent with miR-205 inhibitors, overexpressed CDK2AP1 suppressed the activity of MMP2 and MMP9 and c-Myc and CyclinD1 expression in LSCC cells.

**Conclusion:**

These findings help us to better elucidate the molecular mechanisms of LSCC progression and provide a new theoretical basis to further investigate miR-205 as a potential biomarker and a promising approach for LSCC treatment.

## Background

Laryngeal squamous cell carcinoma (LSCC) is one of the most common cancers with high incidence and mortality [1]. In 2011, LSCCs accounted for approximately 0.7 % of all new cancer diagnoses and leaded to 0.6 % of all cancer-related deaths [2]. Despite improvements of diagnosis and treatment of LSCCs, the survival rate of patients with LSCC has not improved dramatically in the past 20 years [3]. Therefore, it is urgent to elucidate the molecular mechanisms of LSCC proliferation and metastasis, which will provide important insights and help us find new diagnostic and therapeutic approaches to this disease and improve the prognosis of LSCC patients.

MicroRNAs (miRNAs), as a class of small (22-nucleotide) non-coding RNAs, have been identified to be aberrantly expressed in several human malignancies [4]. miRNAs regulate gene expression by binding to the 3′untranslated region (3′-UTR) of their target mRNAs, modulating mRNA stability and/or translation [5]. Previous studies have identified a number of miRNAs that show aberrant expression in LSCC [6–9]. For instance, miR-129-5p is upregulated in primary LSCC tumors and functions as an oncogene in LSCC by repressing APC [8]. miR-139 is down regulated in LSCC tissues and targets CXCR4 and inhibits proliferation and metastasis of LSCC [10]. miR-19a is overexpressed in LSCC and correlated with prognosis and apoptosis of LSCC by regulating TIMP-2 expression [11]. Therefore, further exploration of the expression and function of miRNAs will provide insight into the pathogenesis and progression of LSCC.

Recently, an increasing number of studies have demonstrated that the expression of miR-205 is deregulated in various cancers [12, 13]. For example, downregulated miR-205 expression was observed in breast cancer, and it regulated proliferation and invasion of breast cancer cells by targeting HMGB3 [12]. Significantly lower miR-205 expression levels was also found in prostate cancer tissues and cell lines, and miR-205 exerts tumor-suppressive functions in human prostate through down-regulation of protein kinase cepsilon [14]. miR-205 was also found to be downregulated in esophageal cancer [15]. Meanwhile, recent studies showed the increased expression of miR-205 in lung cancer [16] and endometrial endometrioid carcinoma (EEC) [17]. A significant increase in miR-205 staining intensity was also observed from malignant tumors in head and neck tissue array [18]. Recently, miR-205 was reported to be upregulated in LSCC tissue [19], however, the role and underlying molecular mechanisms of miR-205 in LSCC remains unclear.

Cyclin-dependent kinase 2-associated protein 1 (CDK2AP1), also named as DOC-1 (deleted in oral cancer-1), is a growth suppressor originally isolated from normal hamster oral keratinocytes [20]. There is evidence to suggest that CDK2AP1, as an inhibitor of CDK2 and hence G1/S transition, is expected to involve cell cycle progression [21]. In addition, recent studies showed that CDK2AP1 mediates the growth suppressing signal from TGF-β [22]. Recently, decreased CDK2AP1 expression was shown to correlate with tumor metastasis [23–25] and survival in patients with cancer [26]. CDK2AP1 negatively regulates cell cycle progression and cell proliferation as a target for silencing or downregulation in tumorigenesis. Given the significant association of CDK2AP1 expression with tumorigenesis, CDK2AP1 may play a role in oncogenesis and serves as a molecular target for cancer therapy. However, to date, the specific roles of CDK2AP1 in LSCC have not yet been reported.

The present study showed that miR-205, as an oncogene, was the upstream regulator of CDK2AP1 in LSCC cells. miR-205-mediated CDK2AP1 involves cell proliferation and invasion by increasing the activity of MMP2 and MMP9 and down-regulating *c*-*My*c and *CyclinD1* expression in LSCC cells.

## Results

### miR-205 is negatively associated with CDK2AP1 in clinical LSCC tissues

**Fig. 1:**
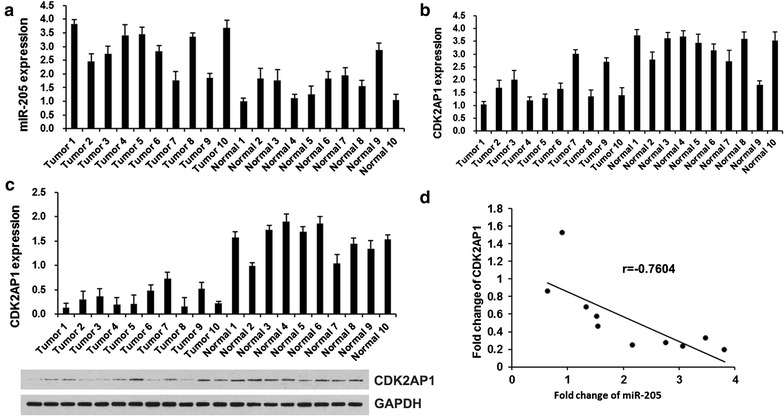
The expression of miR-205 and CDK2AP1 in NPC tissue. **a** miR-205 expression levels was examined by qRT-PCR in 10 cases of clinical LSCC tissues and paired non-tumorous tissues. **b** CDK2AP1 expression levels was examined by qRT-PCR in 10 cases of clinical LSCC tissues and paired non-tumorous tissues. **c** CDK2AP1 expression levels was examined by western blot in 10 cases of clinical LSCC tissues and paired non-tumorous tissues. **d** Correlation of miR-205 levels with CDK2AP1 levels was examined by qRT-PCR in 10 cases of clinical LSCC tissues (Pearson’s correlation coefficient, r = −0.7604)

Next, we analyzed the miR-205 and CDK2AP1 expression in 10 paired clinical LSCC and adjacent noncancerous tissues using qRT-PCR and western blot. When compared with their noncancerous counterparts, significant upregulation of miR-205 (Fig. 1a) and downregulation of CDK2AP1 (Fig. 1b and c) were observed in all the 10 LSCC samples. Then we assessed the correlation between miR-205 and CDK2AP1. As expected, we found that the levels of miR-205 exhibited a significant negative correlation with the levels of CDK2AP1 mRNA (Pearson’s correlation coefficient of −0.7604, p < 0.01) (Fig. 1d). Overall, our finding indicates that the levels of miR-205 are negatively associated with those of CDK2AP1 mRNA in clinical LSCC tissues.

### miR-205 was the upstream regulator of CDK2AP1

**Fig. 2:**
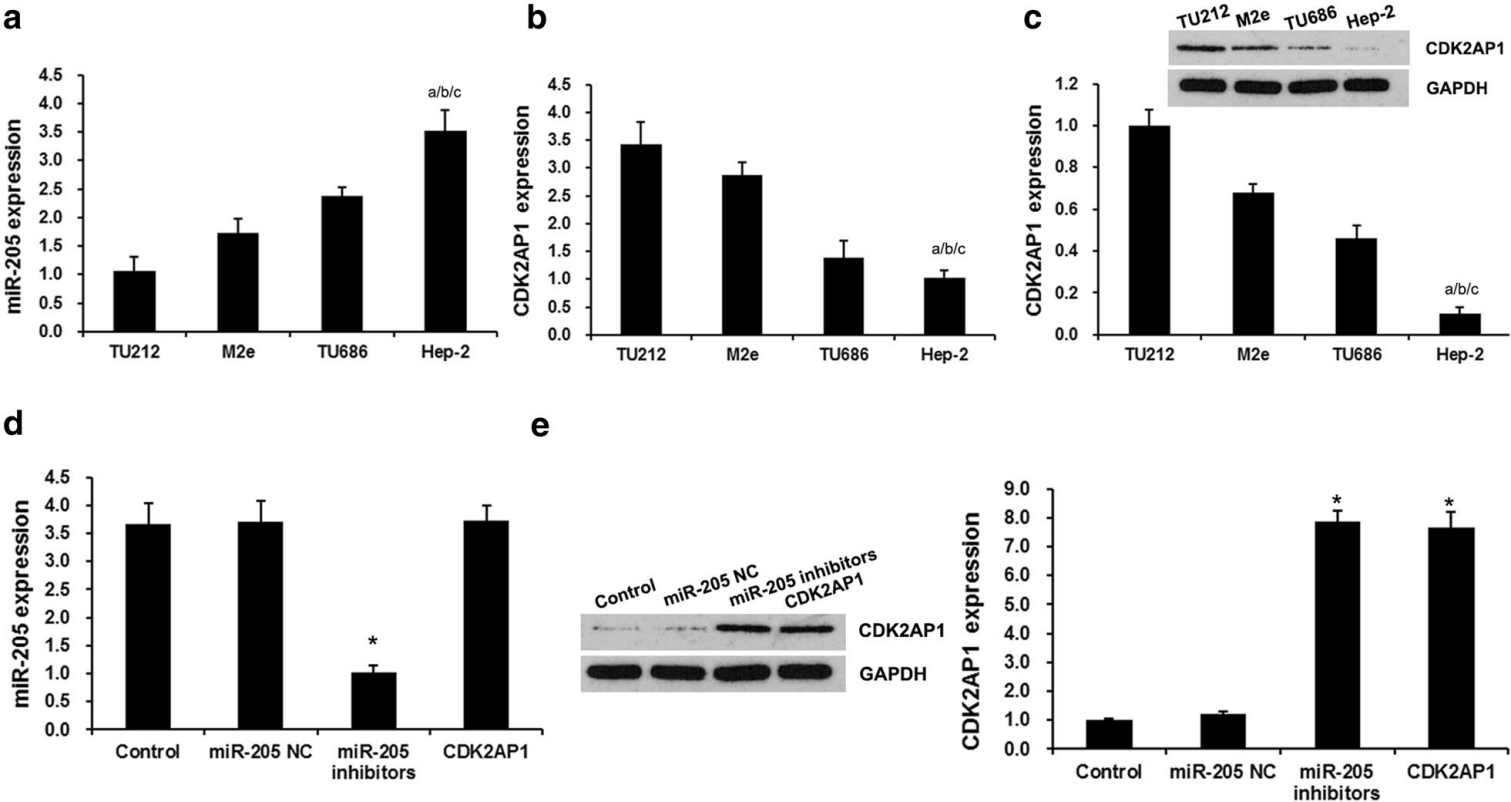
miR-205 was the upstream regulator of CDK2AP1. **a** miR-205 expression in LSCC cell lines. qRT-PCR analysis revealed miR-205 expression in TU212, TU686, M2e and Hep-2 cell lines. *Error bars* represent ± S.E. and ^a^p < 0.01 versus TU212 cells. ^b^p < 0.01 versus M2e cells. ^c^p < 0.01 versus TU686 cells. **b** qRT-PCR analysis revealed CDK2AP1 expression in miR-205 expression in TU212, TU686, M2e and Hep-2 cell lines. **c** Western blot analysis CDK2AP1 expression in miR-205 expression in TU212, TU686, M2e and Hep-2 cell lines.*Error bars* represent ± SD and ^a^p < 0.01 versus TU212 cells. ^b^p < 0.01 versus M2e cells. ^c^p < 0.01 versus TU686 cells. **d** qRT-PCR analysis revealed the effect of miR-205 and CDK2AP1 on miR-205 expression. **e** Western blot analysis revealed the effect of miR-205 and CDK2AP1 on CDK2AP1 expression. Hep-2 cells were transfected with miR-205 (miR-205 group) or miR-205 negtive control (miR-205 NC group) or CDK2AP1 (CDK2AP1 group). *Error bars* represent ± SD and ^*^p < 0.01 versus control group

Previous studies showed that miR-205 was upregulated in LSCC tissue [19]. Here, we detected the expression of miR-205 in four LSSC cell lines (TU212, Hep-2, TU686 and M2e). As shown in Fig. 2a, the real-time PCR assay showed that the expression level of miR-205 was markedly upregulated in Hep-2 cell line compared with other LSSC cell lines (TU212, TU686 and M2e).

The miRNA target prediction websites http://www.microRNA.org and TargetScan showed that CDK2AP1 is the target gene of miR-205. To further confirm CDK2AP1 is a potential downstream target gene of miR-205 in LSCC cells, we detected the expression of CDK2AP1 in four LSSC cell lines (TU212, Hep-2, TU686 and M2e) and the effects of miR-205 on CDK2AP1 expression. As shown in Fig. 2b and c, mRNA expression level of CDK2AP1 was significantly lower in Hep-2 cell line, in comparison with the expression levels in TU212, TU686 and M2e cell lines, which was consistent with protein expression levels of CDK2AP1 using western blot analysis. So Hep-2 cell line was used for the following further analysis.

Furthermore, we silenced miR-205 or overexpressed CDK2AP1 to detect the interaction of miR-205 with CDK2AP1. As shown in Fig. 2d, miR-205 inhibitors silenced miR-205 expression significantly, however, overexpressed CDK2AP1 had no effect on miR-205 expression. Fig 2e showed that miR-205 inhibitors induced CDK2AP1 expression evidently. Taken together, these results indicated that CDK2AP1, as an oncogene, was a potential downstream gene of miR-205 in LSCC.

### miR-205 promotes cell proliferation and invasion by suppressing CDK2AP1 expression in LSCC

**Fig. 3:**
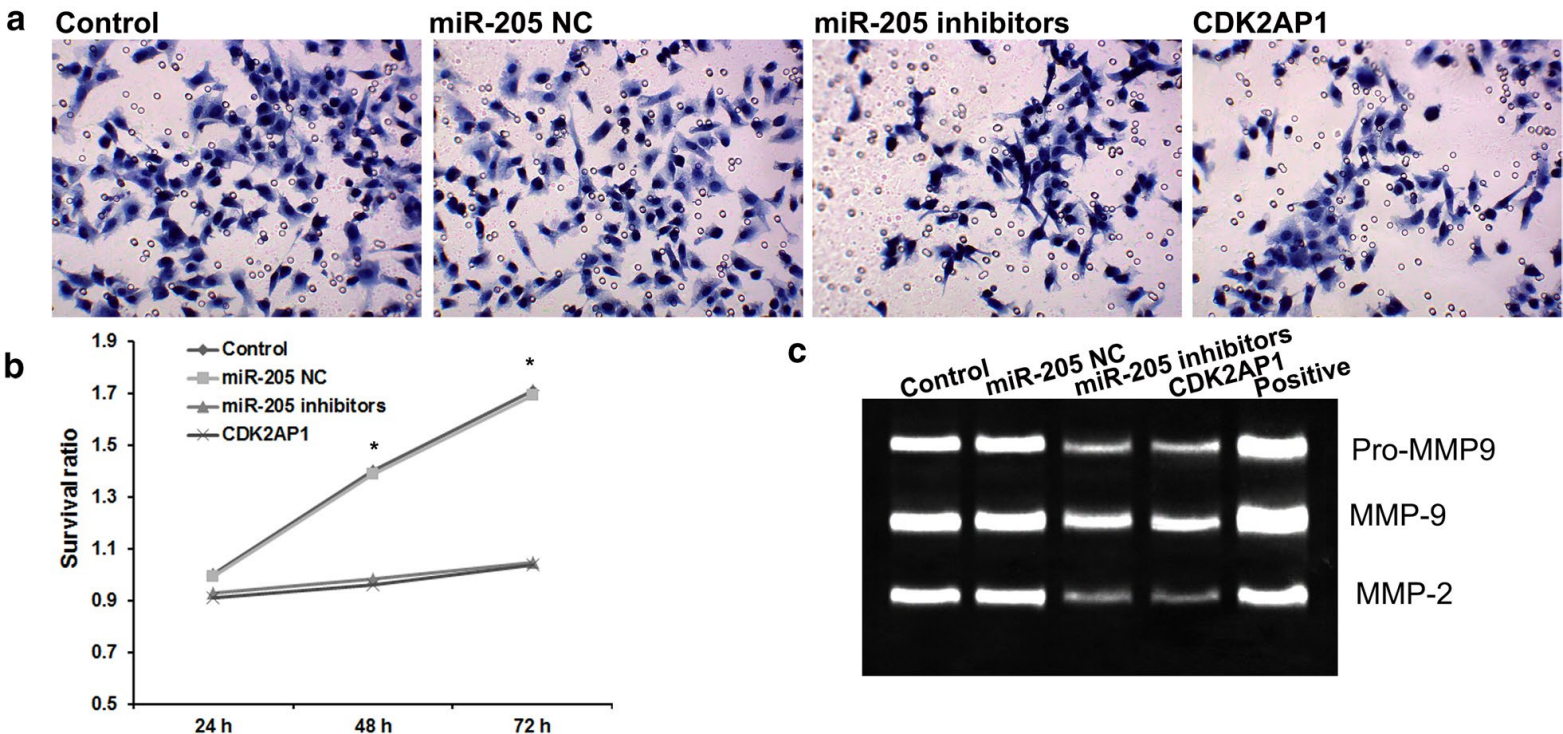
miR-205 promotes cell proliferation and invasion by suppressing CDK2AP1 expression in LSCC. **a** Transwell invasion assay revealed the effects of miR-205 and CDK2AP1 on LSCC invasion. **b** MTT assay revealed the effects of miR-205 and CDK2AP1 on LSCC proliferation. **c** Gelatin zymography assay revealed effects of miR-205 and CDK2AP1 on activity of MMP2 and MMP9 in LSCC

To evaluate the impact of miR-205 on LSCC cell invasion, transwell invasion assays were employed in this study. The results showed that silenced miR-205 decreased the invasion of Hep-2 cells, which was consistent with overexpressed CDK2AP1 (Fig. 3a). Figure 3b showed the effects of miR-205 and CDK2AP1 on the proliferation of LSCC cells using a 3-(4,5-dimethylthiazal-2-yl)-2,5-diphenyl-tetrazolium bromide (MTT) assay. Consistent with miR-205 inhibitors, overexpressed CDK2AP1 significantly decreased the proliferation ability of HCC cells at 48 and 72 h, compared with control group (Fig. 3b).

### miR-205 regulates activity of MMP2 and MMP9 by suppressing CDK2AP1 expression in LSCC

Furthermore, we detected the effects of miR-205 and CDK2AP1 on the activity of MMP2 and MMP9 in Hep-2 cells. As shown in Fig. 3c, the activity of MMP2 and MMP9 was significantly downregulated by miR-205 inhibitors and CDK2AP1 compared to control group. It suggested that miR-205 promotes the activity of MMP2 and MMP9 by suppressing CDK2AP1 expression in LSCC cells.

### miR-205 induces *c*-*Myc* and *CyclinD1* expression by suppressing CDK2AP1 expression

**Fig. 4:**
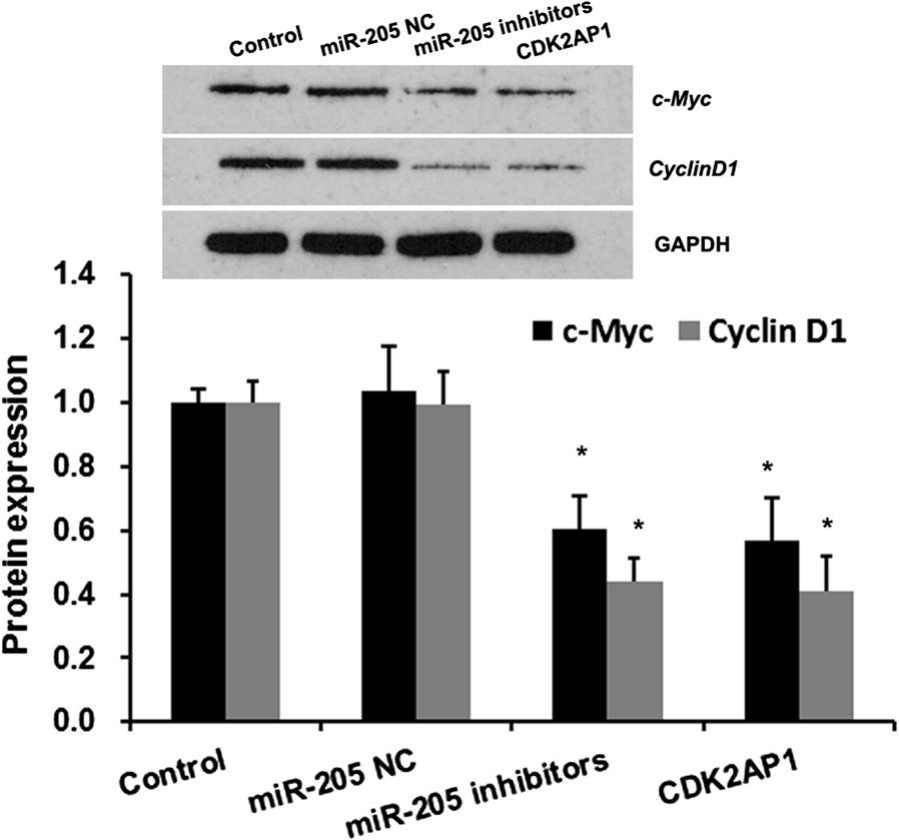
miR-205 induces the expression of *c*-*Myc* and *CyclinD1* by targeting CDK2AP1. Hep-2 cells were transfected with miR-205 mimics (miR-205 group) or miR-205 negative control (miR-205 NC group) or CDK2AP1 (CDK2AP1 group). Forty-eight hours later, proteins were harvested for Western blotting; GAPDH served as an internal control. *Error bars* represent ± SD *p < 0.01 versus control and NC

CDK2AP1 is a cell cycle regulator, it upregulates cell cycle inhibitory genes and suppresses G1-S transition [27]. *c*-*Myc*, and *CyclinD1* are oncoproteins that play an important role in cell proliferation, cell cycle regulation, and invasion, respectively [28, 29]. In the present study, Western blotting was used to investigate whether miR-205 regulates the expression of *c*-*Myc* and *CyclinD1* through suppressing CDK2AP1 expression (Fig. 4). The results revealed that the expression levels of *c*-*Myc* and *CyclinD1* in miR-205 inhibitors group and overexpressed CDK2AP1 group were significantly lower than that in control group. These results provide further support of the inhibitory role that miR-205 plays in the proliferation, invasion of LSCC cells by targeting CDK2AP1.

## Discussion

miR-205 is involved in the genesis and progression of human malignancies. Dysregulation of miR-205 expression was found in various tumors and implicated in the regulation of cell apoptosis, proliferation and metastasis. Several studies have shown that miR-205 is downregulated in different tumor types: Yin et al. found that miR-205 is significantly downregulated in gastric cancer tissues, compared with adjacent normal tissues [30]. miR-205 is also up-regulated in breast cancer [12], prostate cancer [14], esophageal cancer [15] and head and neck carcinomas [31]. In contrast, miR-205 expression has been found to be up-regulated in esophageal squamous cell carcinoma [32]. Recent studies showed the increased expression of miR-205 in lung cancer [16] and endometrial endometrioid carcinoma (EEC) [17]. Although Tian’s work is contradictory and shows that miR-205 expression is downregulated in LSCC [33], Cao et al. confirmed that miR-205 showed higher expression in (LSCC) tissue than in adjacent normal tissues [19], Kimura et al. reported high expression levels of miR-205 in both malignant and benign squamous epithelia [34]. In this study, real-time qPCR and western blot assay showed that expression of miR-205 was upregulated and expression of CDK2AP1 was upregulated in LSCC tissues, and the expression levels of miR-205 were negatively related to those of CDK2AP1 in LSCC tissues. Next, we detected the expression of miR-205 in four LSSC cell lines (TU212, Hep-2, TU686 and M2e) and found that miR-205 expression in Hep-2 cell line was higher compared with other LSSC cell lines (TU212, TU686 and M2e). miR-205 inhibitors suppressed LSCC proliferation and invasion. These results indicated that miR-205, as an oncogene, regulated proliferation and invasion of LSCC.

CDK2AP1, corresponding to the gene doc-1, is an S-phase-associated growth suppressor that binds with DNA polymerase A/primase and/or CDK2 [20, 35]. Recently, emerging evidence suggests that loss or reduced expression of CDK2AP1 might contribute to the tumorigenesis in many types of malignancies including esophageal carcinoma, oral cancer, prostate cancer, colorectal cancer and gastric cancer [22, 25, 36–39]. In this study, we detected the expression of CDK2AP1 in four LSSC cell lines (TU212, Hep-2, TU686 and M2e) and found that expression levels of CDK2AP1 was significantly lowest in Hep-2 cell lines, in comparison with the expression levels in TU212, TU686 and M2e cell lines. miRNAs were found to exert their biological functions by targeting its target genes. Recently, CDK2AP1 was reported as the target gene of miR-21 in malignant human oral keratinocyte [40]. However, no other miRNA was found to be the regulator of CDK2AP1. In this study, CDK2AP1 expression was suppressed by miR-205, indicating that CDK2AP1 is a downstream gene of miR-205 in LSCC. Consistent with other tumors, overexpressed CDK2AP1 also suppressed cell proliferation and invasion in LSCC. Moreover, overexpressed CDK2AP1 reduced the activity of MMP2 and MMP9 in LSCC evidently.

It has been demonstrated that CDK2AP1, as cell cycle regulator, is an important candidate target for therapeutic intervention [41, 42]. *c*-*Myc* and *cyclin D1* are cell cycle regulatory proteins and reported to involve tumorigenesis [43–45]. In this study, attention is also paid to the issues regarding association between the antitumor activity of CDK2AP1 and expression of *c*-*Myc* and *cyclin D1* gene and our finding shows that miR-205 inhibitors and ectopic CDK2AP1 reduced the expression of *c*-*Myc* and *cyclin D1* in LSCC cells.

## Conclusion

Our findings strongly suggest that CDK2AP1 is a downstream gene of miR-205 and facilitates important regulatory roles in cell proliferation and invasion progression of LSCC via *c*-*Myc/cyclin D1* pathway. Hence, this study extends our current knowledge on oncogenesis of LSCC and indicates that CDK2AP1 may serve as a new molecular target for LSCC therapy.

## Methods

### Human tissue specimens

Ten clinical LSCC tissues and their corresponding noncancerous tissues used in this study were obtained from Wuhan Union Hospital of Tongji Medical College (Hubei, China) after surgical resection. Informed consents were obtained from each patient to approve the use of their tissues for research purposes. The study protocol was approved by the Institute Research Ethics Committee at Tongji Medical College.

### Cell culture and transfection

The LSCC cell lines TU212, Hep-2, TU686 and M2e were used in this study. All cells were cultured DMEM medium (Invitrogen, Carlsbad, CA, USA) supplemented with 10 % fetal bovine serum (HyClone, Logan, Utah, USA) and 1 % penicillin/streptomycin and incubated in a humidified (37 °C, 5 % CO_2_) incubator.

The miR-205 inhibitors and negative control molecules were synthesized by GenePharma Co., Ltd. (Shanghai, China), added to culture media at a final concentration of 100 nM and transfected into cells using Lipofectamine™ 2000 (Invitrogen Life Technologies) according to the manufacturer’s instructions.

### Quantitative real-time PCR

Total RNA was isolated from Hep-2 cell lines using Trizol reagent (Invitrogen). The expression of miR-205 was quantified by qRT-PCR using TaqMan microRNA assays (Applied Biosystems, Carlsbad, CA, USA) and normalized to U6B. The expression of CDK2AP1 was quantified by qRT-PCR using SYBR-Green assays (Applied Biosystems) and normalized to β-actin. Gene expression was calculated using the 2^−ΔCt^ method. The primers were as follows: miR-205: 5′-UCCUUCAUUCCACCGGAGUCUG-3′ and miR-205-inhibitors: 5′-CAGACUCCGGUGGAAUGAAGGA-3′; miR-205 NC: forward primer: 5′-UUCUCCGAACGUGUCACGUTT-3′ and reverse primer: 5′-ACGUGACACGUUCGGAGAATT-3′. CDK2AP1, forward primer: 5′-TAGTAGCCGGTTCCCATTTG-3′ and reverse primer: 5′-TTAGGTTTGGTGGGTTGCTC-3′.

### Western blot analysis

Whole cell extracts were prepared with a cell lysis reagent (Sigma-Aldrich, St. Louis, MO, USA) according to the manual, and then, the protein was quantified by a BCA assay (Pierce, Rockford, IL, USA). Then, the protein samples were separated by SDS-PAGE (10 %) and detected by Western blot using polyclonal (rabbit) anti-CDK2AP1, anti-*c*-*Myc* and anti-*cyclinD1* antibody (Santa Cruz Bio-technology, Santa Cruz, CA, USA). Goat anti-rabbit IgG (Pierce, Rockford, IL, USA) secondary antibody conjugated to horseradish peroxidase and ECL detection systems (SuperSignal West Femto, Pierce) were used for detection.

### Cell survival assay

The MTT assay was used to estimate cell viability [46]. Briefly, cells were plated at a density of 1 × 10^4^ cells per well in 96-well plates. After exposure to specific treatment, the cells were incubated with MTT at a final concentration of 0.5 mg/ml for 4 h at 37 °C. After the removal of the medium, 150 mM DMSO solutions were added to dissolve the formazan crystals. The absorbance was read at 570 nm using a multi-well scanning spectrophotometer reader. Cells in the control group were considered 100 % viable.

### Invasion assay

The capability of cell invasion was examined by transwell invasion assay. Cells were cultivated to 80 % confluence on the 12-well plates. Then, we observed the procedures of cellular growth at 72 h. All the experiments were repeated in triplicate. The transwell migration chambers were used to evaluate cell invasion. Then cells invasing cells across the membrane were counted under a light microscope.

### Gelatin zymography

MMP Zymography assay kit (for MMP-2 and MMP-9) (Applygen Technologie, China) was used to detect the activity of MMP2 and MMP9. Protein extracts and positive mixture were mixed with an equal volume of 2X SDS-PAGE non-reducing buffer, and electrophoresed on 8 % SDS polyacrylamide gels containing 2 mg/ml of gelatin. Gels were then washed twice for 30 min in buffer A at room temperature, and incubated for 4 h at room temperature in incubation buffer B. Gels were then stained for 2 h with 0.25 % Coomassie brilliant blue and then destained in destaining buffer (10 % acetic acid and 20 % methanol) for 60 min.

### Statistical analysis

Each experiment was repeated at least three times. Data were shown as mean ± SD and analyzed using SPSS 18.0. Statistical comparisons between groups were analyzed using Student’s *t* test and a two-tailed p < 0.05 was considered to indicate statistical significance.
